# T cell abundance in blood predicts acute organ toxicity in chemoradiotherapy for head and neck cancer

**DOI:** 10.18632/oncotarget.11677

**Published:** 2016-08-29

**Authors:** L. Milena Beschel, Martin Leu, Sybille D. Reichardt, Margret Rave-Fränk, Markus A. Schirmer, Christine Stadelmann, Martin Canis, Hendrik A. Wolff, Holger M. Reichardt

**Affiliations:** ^1^ Institute for Cellular and Molecular Immunology, University Medical Center, Georg-August-University Göttingen, Germany; ^2^ Department of Radiotherapy and Radiooncology, University Medical Center, Georg-August-University Göttingen, Germany; ^3^ Institute of Clinical Pharmacology, University Medical Center, Georg-August-University Göttingen, Germany; ^4^ Institute of Neuropathology, University Medical Center, Georg-August-University Göttingen, Germany; ^5^ Department of Otorhinolaryngology, Head and Neck Surgery, University Medical Center, Georg-August-University Göttingen, Germany; ^6^ University Medical Center, Georg-August-University Göttingen, Germany; ^7^ Present address: Strahlentherapie Radiologie München, Germany

**Keywords:** head and neck squamous cell carcinoma, acute organ toxicity, chemoradiotherapy, T cell, predictive marker

## Abstract

Treatment of head and neck squamous cell carcinoma (HNSCC) by chemoradiotherapy (CRT) often results in high-grade acute organ toxicity (HGAOT). As these adverse effects impair the patients' quality of life and the feasibility of the planned therapy, we sought to analyze immunological parameters in tumor material and blood samples obtained from 48 HNSCC patients in order to assess the potential to predict the individual acute organ toxicity. T cells in the tumor stroma were enriched in patients developing HGAOT whereas levels of soluble factors in the plasma and gene expression in whole blood did not coincide with the occurrence of acute organ toxicity. In contrast, the frequency and absolute numbers of selected leukocyte subpopulations measured in samples of peripheral blood mononuclear cells (PBMCs) directly before the beginning of CRT were significantly different in patients with HGAOT as compared to those without. When we validated several potential markers including the abundance of T cells in a small prospective study with 16 HNSCC patients, we were able to correctly predict acute organ toxicity in up to 81% of the patients. We conclude that analysis of PBMCs by fluorescence-activated cell sorting (FACS) might be a convenient strategy to identify patients at risk of developing HGAOT caused by CRT, which might allow to adapt the treatment regimen and possibly improve disease outcome.

## INTRODUCTION

Treatment of patients with locally advanced head and neck squamous cell carcinoma (HNSCC) consists of a multidisciplinary therapeutic approach including surgery and either radiotherapy (RT) or chemoradiotherapy (CRT) [1, 2]. More recently, the addition of new, targeted tumor therapies to standard treatment regimens has been evaluated in clinical trials [3]. Nonetheless, any combined or intensified therapy that aims to improve prognosis can also increase the risk of acute organ toxicity [4, 5]. For HNSCC, the adverse effects comprise a skin reaction, mucositis, and severe dysphagia [6, 7], all of which diminish the patients' quality of life and are associated with increased treatment expenses due to pain therapy or hospital admission [8]. Importantly, any high-grade acute organ toxicity (HGAOT) can even cause the interruption or cessation of the intended CRT, subsequently resulting in a substantial decrease in local control probability [5]. Because the incidence and severity of acute organ toxicity vary considerably among patients with similar disease characteristics and treatment schedules, the identification of intrinsic risk factors is essential. Previous work described the age, gender, body mass index, level of oral health, neutrophil counts, and kidney or hepatic function as patient-related risk factors [9, 10]. More recently, genetic factors [11, 12] and several inflammation markers [8] have been identified as additional putative risk factors. In animal models, for instance, increased tissue expression of COX-2 [13, 14], pro-inflammatory cytokines such as TNFα, IL-6 and IL-1β, or chemokines like CXCL1 and CCL2 were associated with radiation-induced mucositis [15]. In contrast, data on human non-tumor tissue are scarce, with the exception of a report on mucosal and submucosal leukocyte infiltration at the end of the second radiation treatment week, when focal mucositis usually occurs [16]. In contrast to the more complicated acquisition and use of tumor tissue, peripheral blood is a convenient source of diagnostic material that can be studied by quantitative methods and mirrors many immunological features. Therefore, blood analyses are increasingly used to predict the patients' tumor response. For example, two recent studies showed a coherency between overall survival and the (modified) Glasgow Prognostic Score, or the neutrophil-lymphocyte ratio in the blood of HNSCC patients [17, 18]. Patients with squamous cell carcinoma of the esophagus treated with RT showed an association of IL-2 and IFNγ serum levels with local tumor response [19]. However, up to now only a few studies focused on therapy-associated side effects, such as the above-mentioned ones where pro-inflammatory cytokine levels were associated with an increased likelihood to develop HGAOT [19]. Since this analysis, however, did not refer to cytokine levels at the onset of therapy, its predictive value is limited.

We now postulated that the immune system's individual makeup and potency determines the development of HGAOT in each patient, and that it might therefore be possible to identify predictive markers even before treatment is initiated. To test this hypothesis, we first conducted a post-hoc study to identify predictive markers based on the analysis of PBMCs, tumor material, whole blood, and plasma, followed by a small prospective study to validate them. Our results indicate that it is possible to predict HGAOT based on the analysis of immunological markers such as T cell abundance in blood, which might allow to optimize the multimodal treatment approaches, and thereby improve disease outcome.

## RESULTS

### Retrospective pilot study

**Figure 1:**
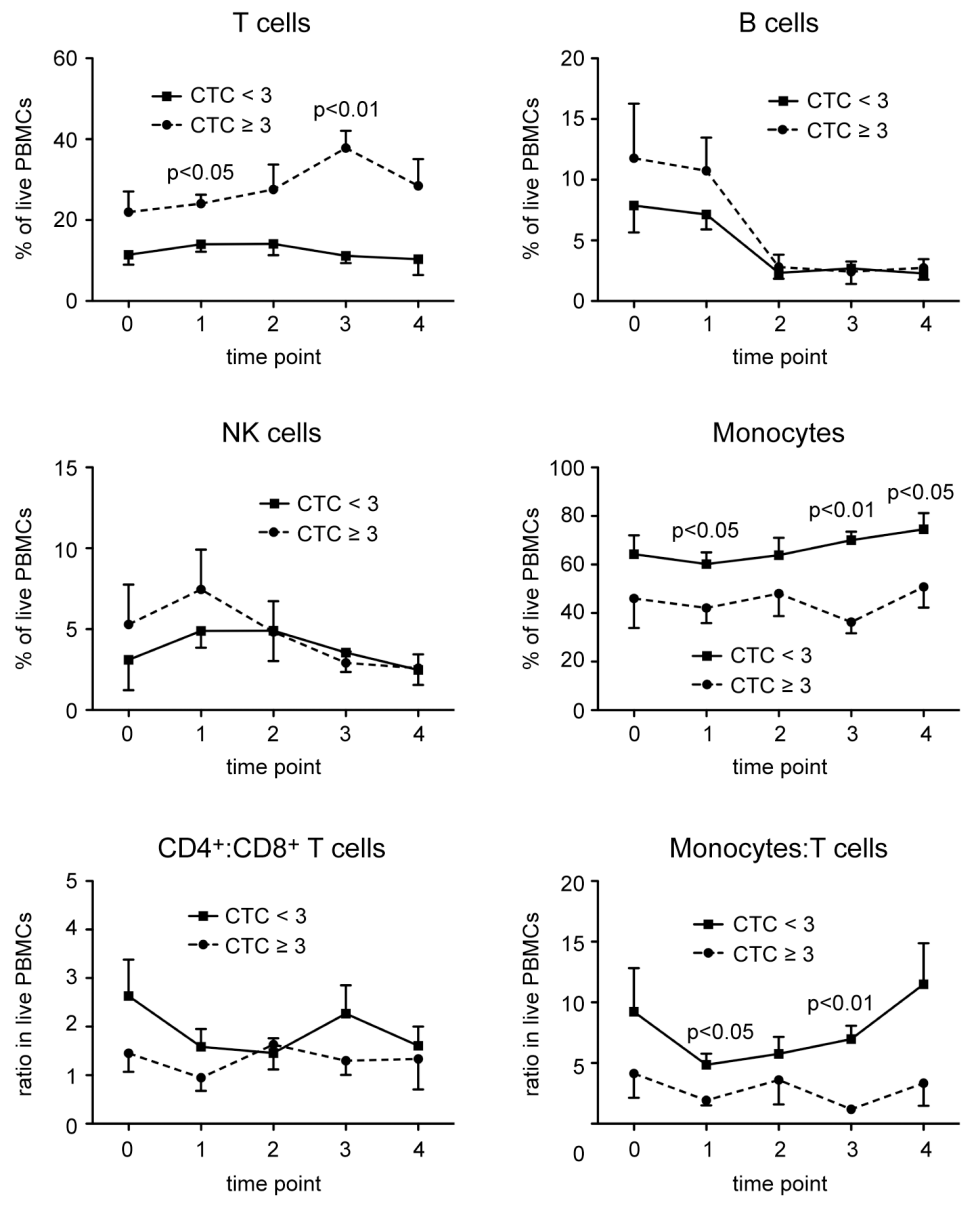
Changes in the frequency of different leukocyte subsets in PBMCs of HNSCC patients in the course of CRT PBMCs were isolated from blood samples obtained six weeks before CRT (0), directly before the beginning (1), in the middle (2), and at the end of CRT (3), and two to three months afterwards during follow-up (4), and stored in liquid nitrogen until use. PBMCs were stained with several combinations of monoclonal antibodies and analyzed by FACS using the gating strategy illustrated in Supplementary Figure S1. The percentages of T cells, B cells, NK cells, and monocytes in live PBMCs, and the ratios between CD4^+^ and CD8^+^ T cells, and between monocytes and T cells in live PBMCs of HNSCC patients without (CTC<3) or with (CTC≥3) HGAOT are shown as the mean ± SEM for each time point during CRT. The solid line refers to patients with CTC<3, the dotted line refers to patients with CTC≥3. N = 6 (CTC<3), N = 7 (CTC≥3). The levels of significance were determined by Mann-Whitney *U* test and are depicted in the graphs; in all other cases the differences were non-significant.

The demographics of all HNSCC patients enrolled in the retrospective study are shown in Table 1. In a pilot study, which included 13 patients, information on the relative abundance of selected immune cell subtypes in PBMCs in relation to the occurrence of HGAOT was obtained at five different time points. Blood was collected six weeks before CRT (0), directly before the beginning (1), in the middle (2), and at the end of CRT (3), and two to three months afterwards during follow-up (4). PBMC samples were stained with different combinations of monoclonal antibodies and analyzed by FACS to determine the percentages of T cells, B cells, NK cells and monocytes. In addition, the ratios between CD4^+^ and CD8^+^ T cells as well as between monocytes and T cells were calculated. Analysis was restricted to the live cell population based on its forward (FSC-A) and side scatter (SSC-A) characteristics, which explains the comparably low proportion of lymphocytes in our samples (Supplementary Figure S1). The acquired parameters showed only moderate changes during the observation period, with the exception of B cell numbers, which markedly dropped after initiation of CRT (Figure 1). In contrast, the chosen immune parameters, in particular the frequencies of T cells and monocytes, differed between patients without or with HGAOT at selected time points, qualifying them as potential predictive markers (Figure 1).

**Table 1:** Patient demographics retrospective study

Characteristic	All patients (N = 48)	
	CTC < 3 N = 21 (43,7%)	CTC ≥ 3 N = 27 (56,3%)
Age, median years +/− sem	63 +/− 11	63 +/− 9
Sex, N (%)		
Male	17 (81)	23 (85.2)
Female	4 (19)	4 (14.8)
**Tumor site, N (%)**		
Oropharynx	10 (47.6)	8^*^ (29.6)
Oral cavity	2 (9.5)	10 (37)
Hypopharynx	5 (23.8)	7^*^ (25.9)
Larynx	4 (19)	3 (11.1)
**Tumor stage, N (%)**		
T1	1 (4.8)	3 (11.1)
T2	4 (19)	8 (29.6)
T3	11 (52.4)	8 (29.6)
T4	5 (23.8)	8 (29.6)
**Nodal stage, N (%)**		
N0	5 (23.8)	3 (11.1)
N1	5 (23.8)	4 (14.8)
N2	11(52.4)	18 (66.7)
N3	0	2 (7.4)
**Histological grading, N (%)**		
1	0	1 (3.7)
2	19 (90.5)	19 (70.4)
3	2 (9.5)	7 (25.9)
**UICC stage, N (%)**		
I	0	0
II	1 (4.8)	1 (3.7)
III	8 (38.1)	5 (18.5)
IV	12 (57.1)	21 (77.8)

* tumor extended from oropharynx to hypopharynx (counted twice)

### Frequency of leukocyte subsets in PBMCs

**Figure 2:**
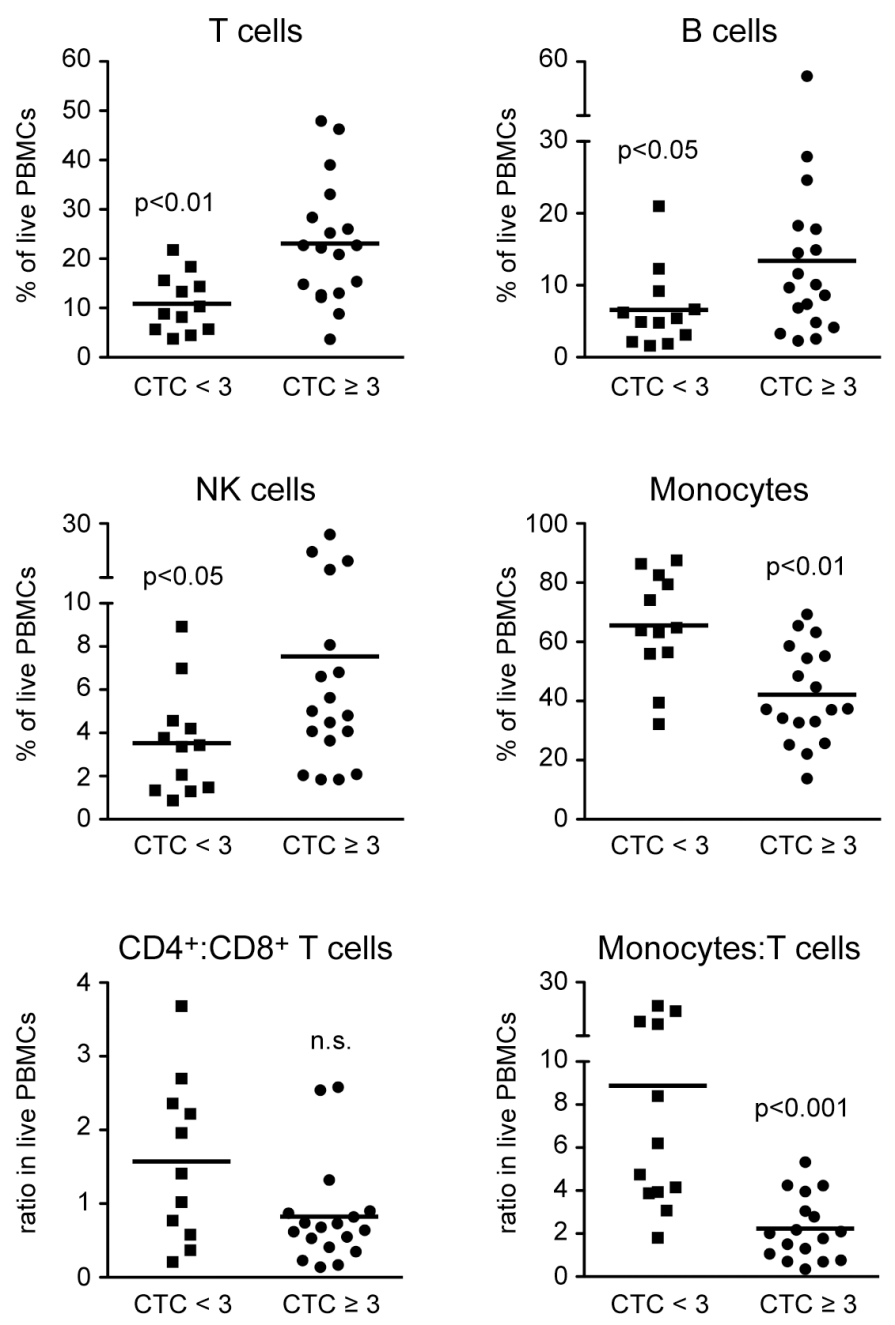
Frequencies of different leukocyte subsets in PBMCs of HNSCC patients directly before the beginning of CRT PBMCs were isolated from blood samples collected directly before the beginning of CRT and stored in liquid nitrogen until use. PBMCs were stained with several combinations of monoclonal antibodies and analyzed by FACS using the gating strategy illustrated in Supplementary Figure S1. The percentages of T cells, B cells, NK cells, and monocytes in live PBMCs, and the ratios between CD4^+^ and CD8^+^ T cells, and between monocytes and T cells are shown for HNSCC patients without (CTC<3) or with (CTC≥3) HGAOT. Each symbol represents one patient, the horizontal line indicates the mean value; N = 11-12 (CTC<3), N = 17-18 (CTC≥3). The levels of significance were determined by Mann-Whitney *U* test and are depicted in the graphs (n.s.: non-significant).

Based on the results of the pilot study, all subsequent FACS measurements were exclusively performed with material obtained directly before the onset of CRT (time point 1). Considering a total of 30 patients of whom good quality PBMC samples were available, we found that the percentages of T cells, B cells and NK cells were increased in the HGAOT group, whereas the percentage of monocytes was concomitantly decreased (Figure 2). Furthermore, the ratio between monocytes and T cells was lower in patients with CTC≥3, but the ratio between CD4^+^ and CD8^+^ T cells was not altered (Figure 2). Collectively, these data indicate that the composition of PBMCs before therapy is closely linked to the development of acute organ toxicity.

### Absolute immune cell numbers in blood

**Figure 3:**
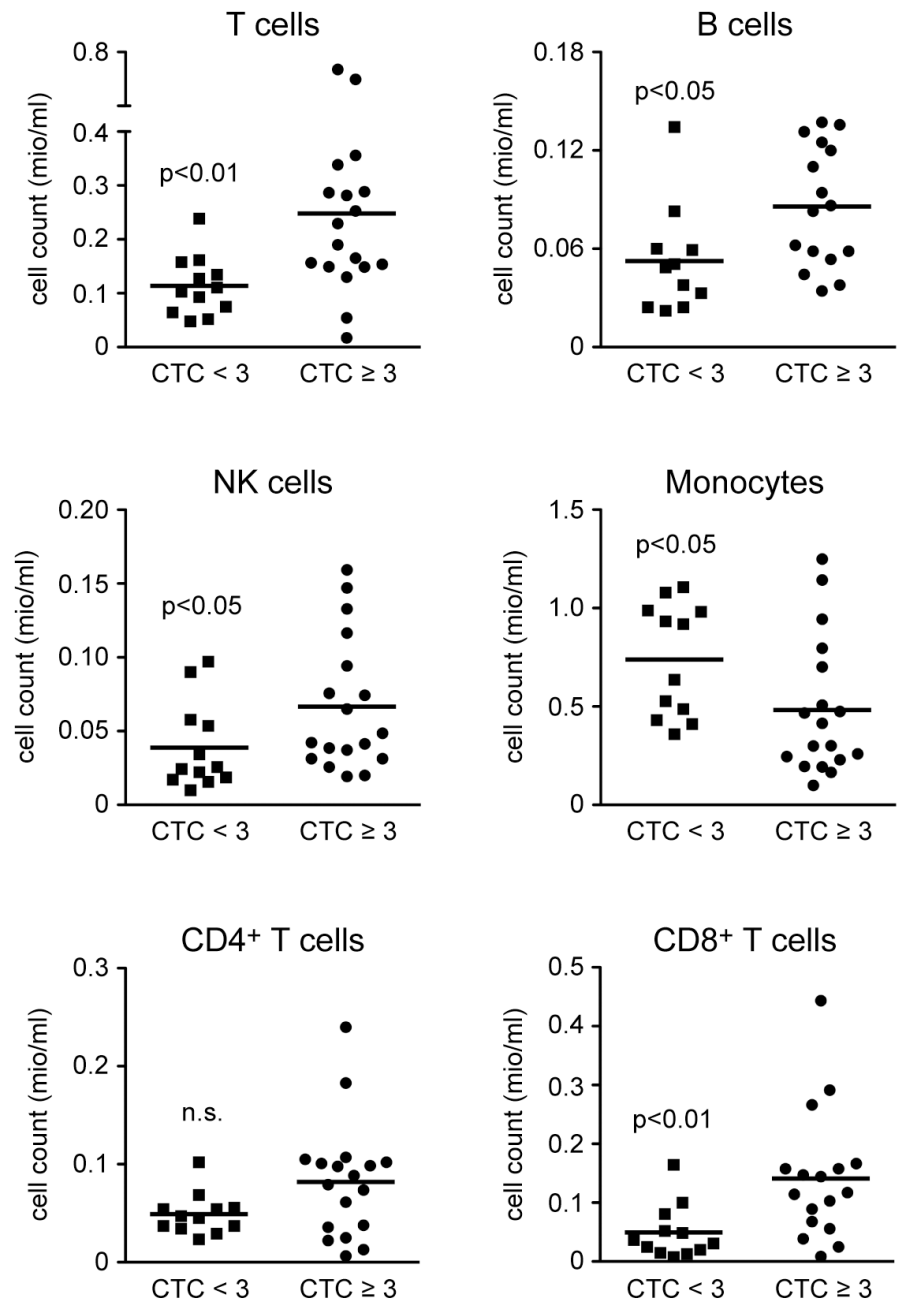
Absolute cell numbers of leukocyte subsets in the blood of HNSCC patients directly before the beginning of CRT PBMCs were isolated from blood samples of a defined volume directly before the begining of CRT and stored in liquid nitrogen until use. Absolute numbers of T cells, B cells, NK cells, monocytes, CD4^+^ T cells, and CD8^+^ T cells were calculated based on the amount of PBMCs obtained from each HNSCC patients without (CTC<3) or with (CTC≥3) HGAOT after isolation by density gradient centrifugation and their individual frequencies as determined by FACS analysis. Cell counts are depicted in million cells per ml of peripheral blood. Each symbol represents one patient, the horizontal line indicates the mean value; N = 11-12 (CTC<3), N = 16-18 (CTC≥3). The levels of significance were determined by Mann-Whitney *U* test and are depicted in the graphs (n.s.: non-significant).

We additionally quantified the absolute numbers of different types of immune cells per ml of peripheral blood and came to similar conclusions as for the corresponding percentages. T cell, B cell and NK cell numbers were higher in patients with HGOAT whereas monocyte numbers were reduced (Figure 3). The absolute numbers of CD8^+^ T cells were significantly higher in the CTC≥3 group, whereas CD4^+^ T cell numbers did not appear to correlate with the occurrence of HGAOT (Figure 3). These findings suggest that immune cell counts in the blood of HNSCC patients are good candidates for predictive markers of treatment-related adverse effects. Of note, tests for an influence of patient age, gender, or applied therapy on immune cell counts did not reveal any associations (data not shown).

### T cell infiltration in pre-treatment tumor material

**Figure 4:**
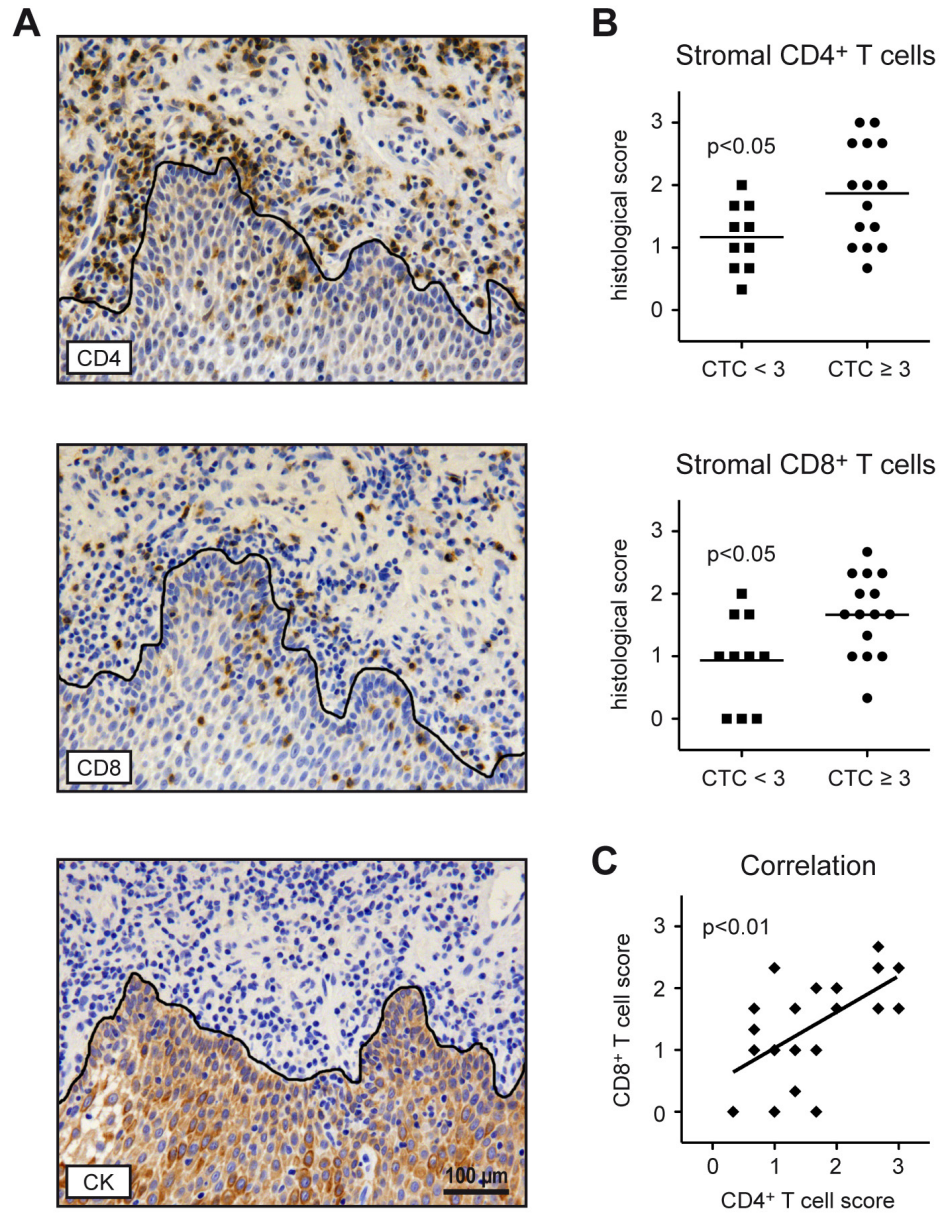
T cell infiltration in pre-treatment tumor material from HNSCC patients Consecutive paraffin sections prepared from biopsies or tumor material collected during surgical resection were stained with antibodies recognizing CD4, CD8 or the epithelial cell marker CK AE 1/3. **A.** Representative photographs of tissue sections stained with each of the three antibodies are shown. Positively stained cells were identified by their dark brown color. The black line delineates the border between the tumor and the surrounding stromal compartment and is based on the CK AE 1/3 staining. Size bar: 100 μm. **B.** Semi-quantitative scoring of CD4^+^ and CD8^+^ T cells in the stroma of tumors from HNSCC patients without (CTC<3) or with (CTC≥3) HGAOT using a 3-point-scale (0 = no T cells present; 1 = weak T cell infiltration; 2 = moderate T cell infiltration; 3 = intense T cell infiltration). Each symbol represents one patient, the horizontal line indicates the mean value. **C.** Correlation between CD4^+^ and CD8^+^ T cell scores. Each symbol represents one patient, the solid line represents the regression curve. N = 10 (CTC<3), N = 15 (CTC≥3) for panels B and C. The levels of significance were determined by Mann-Whitney *U* test in panel B, and by Spearman correlation test in panel C.

Tumor material was obtained from 25 patients, either as a biopsy at the time point of diagnosis or during surgical resection, and analyzed by immunohistochemistry. Staining with an antibody against the epithelial cell marker CK AE 1/3 served to distinguish between the tumor and surrounding stromal components (Figure 4A). Since T cells in the tumor itself were often rare or absent, we limited scoring to the stroma (Figure 4A). Semi-quantitative analysis revealed that the abundance of CD4^+^ and CD8^+^ T cells was significantly higher in patients with HGAOT as compared to those ones with milder therapy-related adverse effects (Figure 4B). Furthermore, the degree of infiltration by both T cell subsets was significantly correlated (Figure 4C). We conclude that the number of T cells *in situ* correlates with the development of HGAOT. Interestingly, when we plotted the scores of CD4^+^ and CD8^+^ T cell infiltration in the tumor stroma against absolute total T cell numbers in the blood (see Figure 3), patients without or with HGAOT fell into two separate, non-overlapping groups that could be completely delineated from each other (Supplementary Figure S2).

### Gene expression analysis of whole blood

**Figure 5:**
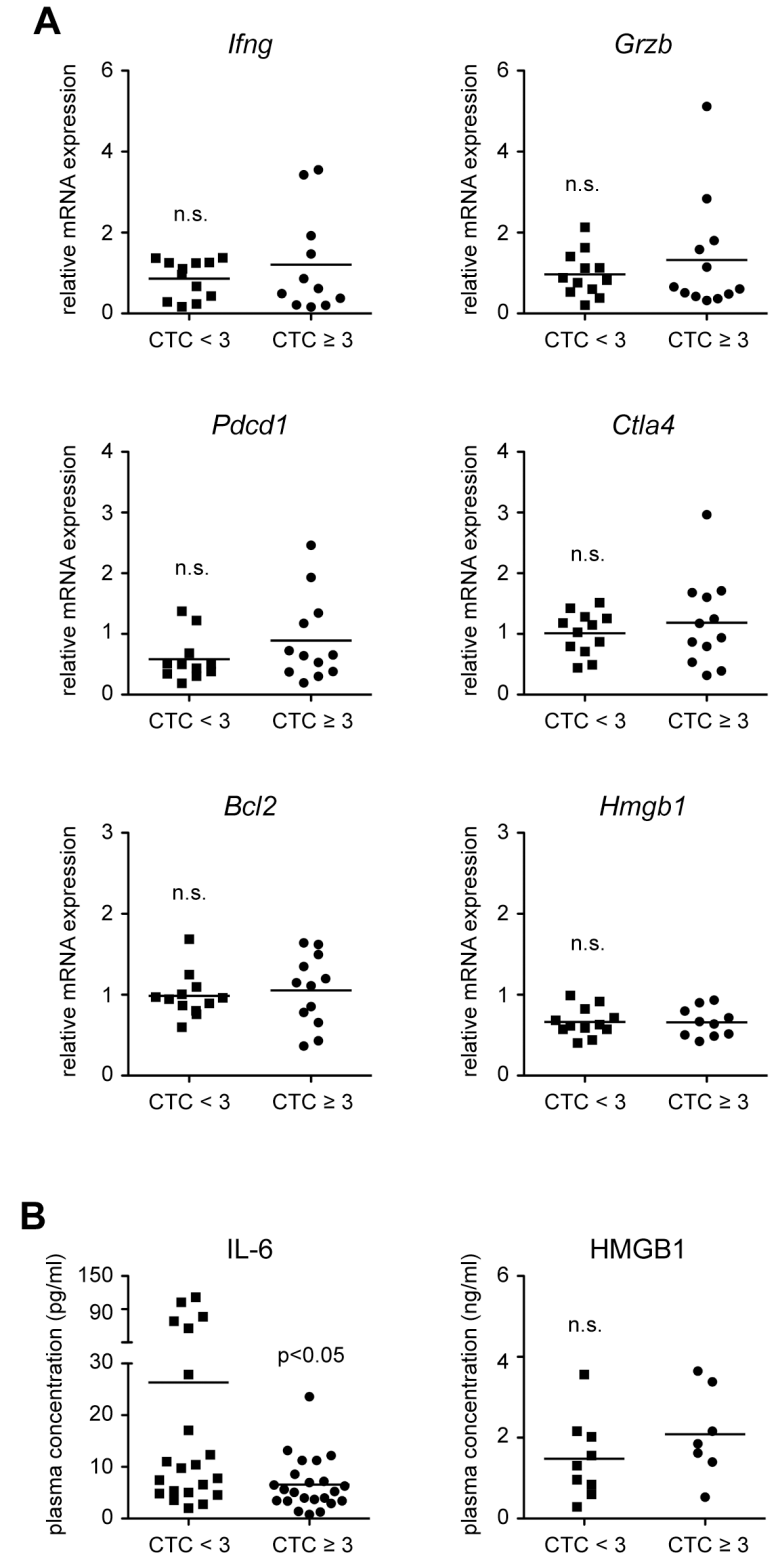
Gene expression in whole blood samples and levels of soluble factors in the plasma of HNSCC patients directly before the beginning of CRT **A.** RNA was isolated from whole blood of HNSCC patients without (CTC<3) or with (CTC≥3) HGAOT directly before the beginning of CRT, transcribed into cDNA and analyzed by quantitative RT-PCR for the relative expression of *Ifng* (*interferon gamma*), *Grzb* (*granzyme B*), *Pdcd1* (*programmed cell death 1*), *Ctla4* (*cytotoxic T lymphocyte associated protein 4*), *Bcl2* (*B cell lymphoma 2*), and *Hmgb1* (*high mobility group box 1*); *Hprt* (*hypoxanthine phosphoribosyltransferase*) was used as a housekeeping gene. Quantification was achieved using the ΔΔCt method. Each symbol represents one patient, the horizontal line indicates the mean value; N = 11-12 (CTC<3), N = 10-12 (CTC≥3). **B.** Plasma was isolated from the blood of HNSCC patients without (CTC<3) or with (CTC≥3) HGAOT directly before the beginning of CRT and analyzed for the levels of IL-6 and HMGB1 by ELISA. The results are depicted as absolute values. Each symbol represents one patient, the horizontal line indicates the mean value; N = 21 (CTC<3), N = 23 (CTC≥3) for IL-6; N = 9 (CTC<3), N = 7 (CTC≥3) for HMGB1. The levels of significance in all panels were determined by Mann-Whitney *U* test and are depicted in the graph (n.s.: non-significant).

To analyze additional immunological markers, we isolated RNA from 24 whole blood samples obtained directly before the onset of treatment (time point 1), and studied them for the expression of T cell-related markers by quantitative RT-PCR (Figure 5A). Candidate genes included *Ifng* (*interferon gamma*) and *Grzb* (*granzyme B*), which are expressed by CD8^+^ T cells and NK cells, and the co-inhibitory molecules *Pdcd1* (*programmed cell death 1*) and *Ctla4* (*cytotoxic T lymphocyte associated protein 4*) known to modulate the strength of T cell responses. In addition, we analyzed gene expression of the anti-apoptotic molecule *Bcl2* (*B cell lymphoma 2*), and the alarmin *Hmgb1* (*high mobility group box 1*), which has been reported to be of predictive value for HNSCC outcome [20]. Importantly, there were no differences between HNSCC patients without or with HGAOT in any of these parameters (Figure 5A). This finding indicates that the expression of the analyzed genes before the onset of treatment does not allow to predict HGAOT.

### Cytokine and HMGB1 plasma levels

Earlier studies had indicated that soluble factors might be suitable predictive markers for the clinical outcome of HNSCC or its association with HGAOT [17–20]. It is against this background that we analyzed the levels of IL-2, IFNγ, IL-6 and TNFα in 44 plasma samples obtained directly before the onset of treatment (time point 1) by ELISA. IL-6 was significantly reduced in patients with CTC≥3 as compared to those ones experiencing milder adverse effects (Figure 5B). In contrast, plasma levels of the other three cytokines were very low at the beginning of CRT and not reliably detectable (data not shown). In agreement with our previously obtained gene expression data (see Figure 5A), plasma levels of HMGB1 were similar in both groups (Figure 5B). Collectively, the analysis of soluble factors in the blood is of limited predictive value with regard to the development of HGAOT.

### Prospective validation study

Based on the superior predictive power of the abundance of immune cells in the blood as compared to gene expression in whole blood or the concentration of soluble factors in the plasma, markers were defined that might allow to predict whether patients will develop HGAOT or not. To this end, we selected six parameters and tested them in a small prospective study comprising 16 HNSCC patients. The demographics for this study are provided in Table 2. It is noteworthy that, until the end of the six weeks period of CRT, half of the enrolled patients had developed HGAOT whereas the others had not. We evaluated the percentages of T cells and monocytes, their ratio in PBMCs, and the absolute numbers of T cells, monocytes and CD8^+^ T cells per ml of peripheral blood. The cut-off values that we applied are depicted in Table 3. Depending on each respective factor, the occurrence of HGAOT was correctly predicted in 62-81% of the patients (Table 3). Notably, the absolute numbers of total T cells and CD8^+^ T cells in the blood were the two best single predictive factors amongst the six chosen parameters (Table 3). Taken together, FACS analysis of PBMCs is a promising approach to make predictions as to whether HNSCC patients will develop HGAOT or not.

**Table 2:** Patient demographics prospective study

Characteristic	All patients (N = 16)	
	CTC < 3 N = 8 (50%)	CTC ≥ 3 N = 8 (50%)
Age, median years +/− sem	60 +/− 10	65 +/− 12
Sex, N (%)		
Male	6 (75)	8 (100)
Female	2 (25)	0
**Tumor site, N (%)**		
Oropharynx	3 (37.5)	6 (75)
Oral cavity	1 (12.5)	1 (12.5)
Hypopharynx	1 (12.5)	1 (12.5)
Larynx	2 (25)	0
CUP	1 (12.5)	0
**Tumor stage, N (%)**		
T1	1 (12.5)	0
T2	2 (25)	1 (12.5)
T3	3 (37.5)	3 (37.5)
T4	1 (12.5)	3 (37.5)
Tx	1 (12.5)^*^	1 (12.5)^**^
**Nodal stage, N (%)**		
N0	2 (25)	3 (37.5)
N1	2 (25)	1 (12.5)
N2	4 (50)	4 (50)
N3	0	0
**Histological grading, N (%)**		
1	0	0
2	6 (75)	8 (100)
3	2 (25)	0
**UICC stage, N (%)**		
I	0	0
II	0	0
III	4 (50)	6 (75)
IV	4 (50)	2 (25)

* CUP (Cancer of Unknown Primary),

** RT for nodal recurrence only

**Table 3:** Assessment of predictive factors in a prospective study

Predictive factor	Cut-off value for CTC<3	Correct prediction, N (%)
T cells, relative	< 22%	11 (68.8)
Monocytes, relative	> 38%	11 (68.8)
Monocytes:T cells, ratio	> 1,78	11 (68.8)
T cells, absolute	< 0,25 mio/ml	13 (81.3)
Monocytes, absolute	> 0,33 mio/ml	10 (62.5)
CD8^+^ T cells, absolute	< 0,1 mio/ml	13 (81.3)

## DISCUSSION

This study shows for the first time that differences in immunological parameters before the onset of treatment are closely connected to the development of acute organ toxicity during CRT of locally advanced stages of HNSCC, and that they can serve as predictive markers of HGAOT.

Adverse effects of CRT such as mucositis, dysphagia and a skin reaction are dose-limiting factors in CRT [21]. They result from ionizing radiation and cytotoxic drugs that damage the basal epithelium which subsequently starts to ulcerate [22]. Damaged cells are generated from the tumor as well as neighboring healthy tissue, which causes the secretion of cytokines and chemokines and leads to the local attraction of T cells, monocytes, and neutrophils. Experimental findings revealed that RT stimulates the production of several pro-inflammatory cytokines [23], enhances MHC class I expression [24], and increases susceptibility to T cell recognition and killing. CD8^+^ T cells are especially important in this context since their depletion was found to compromise the efficacy of RT [25, 26].

The analysis of T cell abundance in tissue samples is technically challenging. Biopsies are not always representative of the entire tissue of interest and the scoring system is difficult to standardize. Moreover, the density of T cells was described as being heterogenous within a tumor section, and to vary among individual tumors [27]. Balermpas and colleagues reported on a strong infiltration of the tumor and stroma, as well as on an infiltration of only the stroma or the tumor compartment [27]. In addition, they described differences in the prognostic value of T cell infiltration, depending on the compartment. In contrast, peripheral blood can be studied by quantitative methods such as RT-PCR, ELISA and FACS. Since T cell numbers in the tumor stroma and immune cell abundances in blood both correlated with the occurrence of HGAOT, we expected that gene expression and plasma concentrations of immunological parameters were linked to the development of adverse effects during CRT as well. However, this was not the case despite the fact that all factors that we tested had previously been implicated in T cell activity, inflammation or tumor progression. For instance, IFNγ serum levels in esophageal cancer were reported to correlate with acute organ toxicity [19], whilst we were unable to reliably detect them at all at the onset of CRT. Similarly, an upregulation of HMGB1 had been proposed to predict effective T cell reponses [20], but in our study neither gene expression nor plasma levels of HMGB1 were associated with the development of HGAOT. The only significant finding we made in this respect concerned IL-6 plasma levels. The fact that this cytokine is mainly produced by myeloid cells might explain why patients with HGAOT and a supposedly strong T cell response have lower concentrations of this cytokine in their blood. However, due to its high variability we do not recommend considering it as a predictive factor for HGAOT.

The most promising parameters, which can be easily measured in blood, are the frequencies of several leukocyte subpopulations. FACS analysis revealed that the abundance of T cells, B cells, NK cells, and monocytes in PBMCs correlated with HGAOT. Patients with a high percentage of any of the three former cell types were prone to develop treatment-related side effects whereas the frequency of monocytes was inversely linked to acute organ toxicity. A similar finding was made for the monocyte to T cell ratio, as well as for the absolute number of five different types of immune cells. To our knowledge, this is the first report revealing that frequencies and numbers of T cells and other leukocyte subsets before the onset of treatment significantly correlate with therapy-related side effects.

There are a few other studies that previously assessed the suitability of peripheral blood to obtain predictive markers of acute organ toxicity, including the analysis of chromosomal damage and micronuclei induction in blood lymphocytes [28–30]. In addition, the sensitivity of peripheral blood T cells to apoptosis induction by *in vitro* irradiation was linked to treatment-related toxicity in different forms of cancer [31, 32]. Although this assay appears useful it has some drawbacks. Apoptosis induction is age-dependent, immune cells are manipulated *in vitro*, and the mechanism behind the observed coherency is unknown. In contrast, the results shown in the present work are based on pre-treatment blood samples and are thus not dependent on any therapy-related damage induction.

Earlier investigations had revealed a coherency between HGAOT and prognosis [4, 33, 34], suggesting that the same immunological mechanisms might be responsible for the adverse effects on the one hand and the anti-tumor response on the other hand. The assumption of common traits in the development of toxicity and tumor response is supported by recent data, which indicate a correlation between CD8^+^ T cell infiltration and patient survival [35]. We speculate that the general makeup of the immune system could represent a link between both activities, and that patients developing severe acute organ toxicity have an immune system that is sufficiently potent to cause a strong anti-tumor response as well. We therefore hypothesize that the abundance of T cells in blood mirrors the immune system's overall strength, which presumably impacts acute organ toxicity, T cell infiltration into the tumor, and the patients' overall survival.

Every post-hoc study requires independent validation. As a first step in this direction we tested the six most promising predictive markers in a small prospective study. Importantly, the two best parameters, namely the absolute numbers of total T cells and CD8^+^ T cells, allowed to correctly assign HNSCC patients in 81% of all cases to the two groups either developing HGAOT or not. This result reconfirms that the analysis of T cells in PBMCs is a promising strategy to predict acute organ toxicity during CRT. Undoubtedly, our analyses require further independent validation in a larger prospective study, which should not only aim to test the coherency of blood T cell markers with HGAOT but also their direct association with patient survival.

## MATERIALS AND METHODS

### Ethics statement

Investigation has been conducted in accordance with the ethical standards and according to the *Declaration of Helsinki* and according to national and international guidelines and has been approved by the local ethics committee of the University of Göttingen Medical Center. Informed written consent was obtained from each subject prior to the collection of blood and tumor material.

### Patient characteristics and treatment modalities

A total of 48 patients receiving postoperative CRT after curative surgery for locally advanced HNSCC were included in the retrospective study (Table 1), and a total of 16 patients with similar clinical characteristics were included in the prospective study (Table 2). Following surgery, an integrated intensity-modulated RT was applied daily, five times per week, with single fractions of 2.08 Gy up to 62.4 Gy to the primary tumor area, 1.92 Gy up to 57.6 Gy to the involved lymph nodes, and single fractions of 1.8 Gy up to 54.0 Gy to the drainage sites on both sides of the neck. The vast majority of patients additionally received concomitant low-dose (6 mg/m^2^/TBSA/d, i.v.) or high-dose (40 mg/m^2^/TBSA/d, i.v.) cisplatin combined with facultative anti-emetic medication on each RT day [36]. Biopsies were taken at the time point of diagnosis; additionally, tumor material was collected during surgical resection. Peripheral blood was drawn directly before, at different time points during, and after the treatment. The maximal grade and the onset of organ toxicity comprising a skin reaction, a mucositis and dysphagia were assessed weekly during CRT and every second week following therapy until disappearance according to the *Common Toxicity Criteria for Adverse Events* (CTCAE) [37]. Due to a significant impairment of the quality of life, patients were considered to suffer from HGAOT in the case of a *Common Toxicity Criteria* (CTC) grade 3/4 toxicity for at least one of the scored parameters. In the framework of the present study, grade 3/4 dysphagia was the prevailing adverse event, and it was observed in 24 (50%) patients. Severe mucositis (2 patients) or a severe skin reaction (1 patient) were less frequent. Concomitant treatment with cisplatin in addition to RT had no effect on the occurrence of HGAOT (data not shown).

### Preparation of PBMCs

PBMCs were isolated from 14 ml heparinized whole blood by gradient centrifugation in Biocoll Separating Solution (Biochrome, Berlin, Germany) at 4°C. Buffy coats were collected and washed once in PBS. After counting, PBMCs were incubated in RPMI 1640 medium (Life Technologies, Darmstadt, Germany) supplemented with 10% FCS (Biochrome) at a concentration of 1×10^6^ PBMCs/ml for 24 hours at 37°C. Storage in liquid nitrogen was done in RPMI 1640 medium with 50% FCS and 10% DMSO in a total volume of 1 ml (Sigma, Taufkirchen, Germany).

### FACS analysis

Frozen PBMC samples were quickly defrosted in a water bath at 37°C and resuspended in 10 ml PBS. After centrifugation for 7 min at 350 g, cells were resuspended in PBS plus 0.1% BSA (Sigma) and immediately used for staining with different combinations of fluorochrome-conjugated monoclonal antibodies (BioLegend, Uithoorn, Netherlands): CD3ε (clone: HIT3a, conjugation: APC), CD4 (clone: OKT4, conjugation: FITC), CD8α (clone: HIT8a, conjugation: PE), CD14 (clone: HCD14, conjugation: PE/Cy7), CD19 (clone: HIB19, conjugation: PE/Cy5), CD16 (clone: 3G8, conjugation: APC/Cy7), CD56 (clone: HCD56, conjugation: PE), HLA-DR (clone: L243, conjugation: Alexa488). A FACSCanto II device (BD Biosciences, Heidelberg, Germany) and FlowJo software (Tree Star, Ashland, OR) were used for analysis [38]. Cell viability, i.e. the percentage of cells located in the live gate, was similar in samples from patients without or with HGAOT (CTC<3: 38.3 ± 5.6%; CTC≥3: 41.5 ± 12.2%).

### Immunohistochemistry

The tissue was fixed in 4% PFA, embedded in paraffin, and 2 μm sections prepared according to standard protocols. Immunohistochemical stainings were performed using rabbit-anti-CD4 (clone SP35; Zytomed Systems, Berlin, Germany; 1:100), mouse-anti-CD8 (clone C8/144B; Dako, Glostrup, Denmark; 1:50), or mouse-anti-cytokeratin (clones AE1/AE3; Dako; 1:50) antibodies. Sections were pretreated by cooking at pH=6.0 (citrate buffer, 10 mM; CD8 and AE1/3) or pH=8.0 (EDTA; CD4). Biotinylated secondary antibodies were applied at a dilution of 1:200 (GE Healthcare, Little Chalfont, UK and Jackson Immunoresearch, West Grove, USA). Sections were developed after incubation with extravidin-peroxidase (Sigma; 1:1000) and diaminobenzidine (Sigma), following standard protocols. Nuclei were counterstained with hematoxylin, and sections mounted with DPX (Sigma).

Tumor borders in slides immunostained for CD4 and CD8 were defined on the basis of cytokeratin-positivity on consecutive sections. The level of T cell infiltration in the tumor stroma was evaluated by three independent investigators after taking pictures with an Olympus BX51 microscope at a 200-fold magnification and scored as: 0 (absent), 1 (weak), 2 (moderate) and 3 (intense). In the case of disparate assessments, a mean score was calculated.

### RNA isolation and quantitative RT-PCR

Total RNA was isolated from whole blood specimens collected in PAXgene Blood RNA Tubes (PreAnalytiX, Hembrechtikon, Switzerland). RNA preparation was performed with the PAXgene Blood RNA Kit (Qiagen, Hilden, Germany) according to the manufacturer's instructions. Total RNA content was quantified spectrophotometrically and 1 μg per sample was subjected to reverse transcription using Superscript reverse transcriptase II (Invitrogen, Karlsruhe, Germany) and hexanucleotides (Roche, Mannheim, Germany) as random primers.

Quantitative RT-PCR was performed on an ABI 7500 instrument (Applied Biosystems, Darmstadt, Germany) using the SYBR mastermix from the same company. Results were normalized to mRNA expression of *Hprt* and evaluated using the ΔΔCt method. Primer sequences: *Ifng* (5′-CTGTTACTGCCAGGACCCAT-3′, 5′-TCTGTCACTCTCCTCTTTCCA-3′), *Grzb* (5′-GATG CAGGGGAGATCATCGG-3′, 5′-CTCGTATCAGGA AGCCACCG-3′), *Pdcd1* (5′-CAGTTCCAAACCC TGGTGGT-3′, 5′-GGCTCCTATTGTCCCTCGTG-3′), *Ctla4* (5′-TACCCACCGCCATACTACCT-3′, 5′-GGCA CGGTTCTGGATCAATTA-3′), *Bcl2* (5′-AGATTGAT GGGATCGTTGCCT-3′, 5′-AGTCTACTTCCTCTGTG ATGTTGT-3′), *Hmgb1* (5′-AGAGCGGAGAGA GTGAGGAG-3′, 5′-ATGTTTAGTTATTTTTCCTCAGCGA-3′), *Hprt* (5′-CCTGGCGTCGTGATTAGTGA-3′, 5′-CGAGCAAGACGTTCAGTCCT-3′). All primers were synthesized by Metabion (Planegg, Germany).

### ELISA

Plasma was isolated from venous blood drawn into EDTA-treated tubes. Concentrations of soluble factors were measured using commercially available ELISA kits for IL-2, IFNγ, IL-6 and TNFα (Biolegend, Uithoorn, The Netherlands) as well as HMGB1 (IBL International, Hamburg, Germany) according to the manufacturers' instructions.

### Statistical analysis

Data were analyzed using Prism^®^ software (GraphPad, San Diego, CA). The Mann-Whitney *U* test and the Spearman correlation test were applied to analyze whether immunological parameters were significantly different between both groups of patients.

## SUPPLEMENTARY MATERIALS FIGURES


