# Supplementary material for: T cell abundance in blood predicts acute organ toxicity in chemoradiotherapy for head and neck cancer
Source: Oncotarget. 2016 Aug 29;7(40):65902–15. doi: 10.18632/oncotarget.11677 (PMC5323201; doi:10.18632/oncotarget.11677)
Supplement: Supplementary file 1 [file oncotarget-07-65902-s001.pdf]

# T cell abundance in blood predicts acute organ toxicity in chemoradiotherapy for head and neck cancer

## SUPPLEMENTARY FIGURES

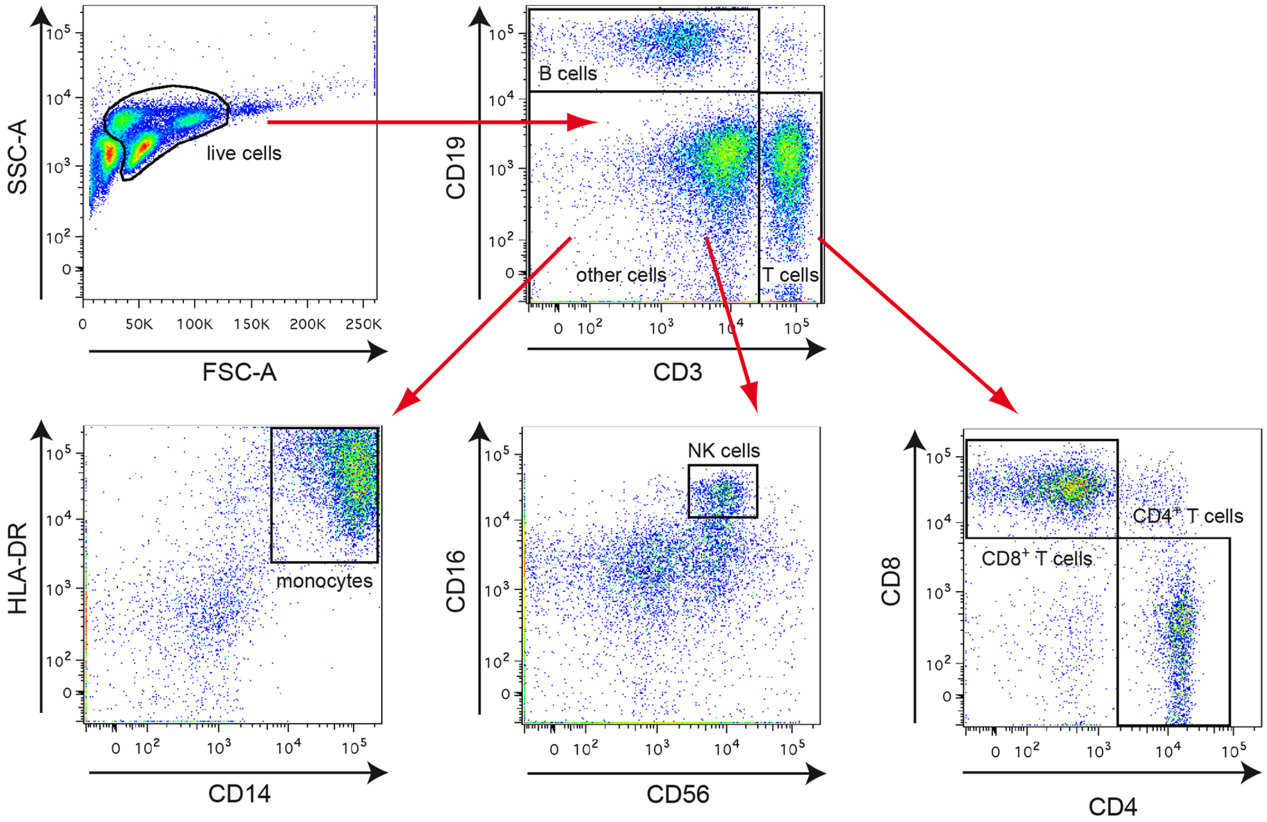


**Supplementary Figure S1: Gating strategy for the analysis of PBMCs by FACS.** The analysis was done with PBMCs obtained from a representative patient with HGAOT at time point 1, i.e. directly before the beginning of CRT. Live cells in PBMCs were defined on the basis of their forward (FSC-A) and side scatter (SSC-A) characteristics. Live cells were further subdivided into B cells based on CD19 expression, T cells based on CD3 expression, and other cells being negative for both markers. Monocytes were defined by concomitant expression of CD14 and HLA-DR, and NK cells were defined by concomitant expression of CD16 and CD56. T cells were further subdivided into $CD4^+$ and $CD8^+$ T cells according to the expression of the respective surface molecules.

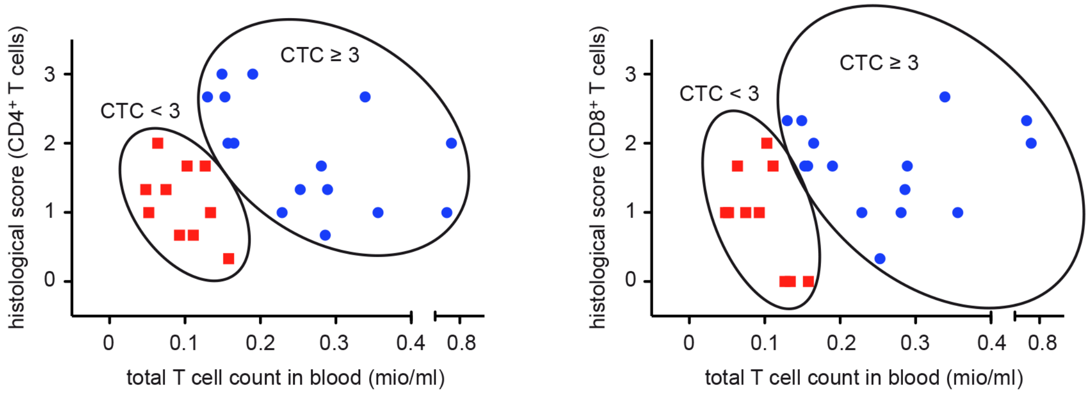


**Supplementary Figure S2: Correlation between T cell infiltration in pre-treatment tumor material from HNSCC patients and absolute T cell numbers in the blood directly before the beginning of CRT.** The semi-quantitative scoring of $CD4^+$ T cells (left panel) or $CD8^+$ T cells (right panel) in the stroma of tumors from HNSCC patients without (CTC<3) or with (CTC≥3) HGAOT using a 3-point-scale (see Figure 4) is depicted on the y-axis. The corresponding absolute total T cell numbers per ml of peripheral blood as determined by FACS analysis (see Figure 3) are depicted on the x-axis. Each symbol represents one individual patient; N = 10 (CTC<3), N = 15 (CTC≥3). Patients with CTC<3 (red squares) or CTC≥3 (blue circles) fall into two separate groups indicated in the graphs.
